# CD4 T-Cell-Independent Antibody Response Reduces Enterovirus 71 Lethality in Mice by Decreasing Tissue Viral Loads

**DOI:** 10.1155/2012/580696

**Published:** 2012-10-24

**Authors:** Li-Chiu Wang, Chia-Min Kao, Pin Ling, Ih-Jen Su, Tung-Miao Chang, Shun-Hua Chen

**Affiliations:** ^1^Institute of Basic Medical Sciences, Medical College, National Cheng Kung University, Tainan 701, Taiwan; ^2^Department of Microbiology and Immunology, Medical College, National Cheng Kung University, Tainan 701, Taiwan; ^3^National Institute of Infectious Diseases and Vaccinology, National Health Research Institutes, Tainan 701, Taiwan; ^4^Statistical Analysis Laboratory, Department of International Business Management, Tainan University of Technology, Tainan 701, Taiwan; ^5^Center of Excellence for Infectious Diseases and Signaling Research, National Cheng Kung University, Tainan 701, Taiwan

## Abstract

Enterovirus 71 (EV71) has induced fatal encephalitis in hundreds of thousands of infants and young children in the Asia-Pacific region since the past decade. Lymphocyte and antibody responses have been suspected to aggravate EV71-induced neurological symptoms, so anti-inflammatory agents have been used to treat patients with neurological symptoms. In the present study, we found that mice deficient in CD4^+^ T cells were resistant to EV71 infection as wild-type mice, whereas mice deficient in B cells were highly susceptible to viral infection. Compensation of CD4 T-cell function by other immune cells was not likely, because wild-type mice depleted of CD4^+^ T cells were also resistant to viral infection. Infected CD4 T-cell-deficient mice produced virus-specific neutralizing antibodies, IgM and IgG. Moreover, adoptive transfer of the virus-specific antibody produced by infected CD4 T-cell-deficient mice protected B-cell-deficient mice from infection by reducing tissue viral loads. Collectively, our results show that the CD4 T-cell-independent antibody response promotes the survival of EV71-infected mice and suggest great potential for the use of vaccines and neutralizing antibodies to reduce fatal symptoms in patients.

## 1. Introduction 

Enterovirus 71 (EV71), a member of the family *Picornaviridae*, can infect the human central nervous system (CNS) to induce fatal neurological manifestations [1–3]. Severe symptoms, brainstem encephalitis with cardiopulmonary complications, often induce death or long-term neurological sequelae, particularly in young children. EV71 outbreaks have been estimated to cause hundreds of cases of death and thousands of cases of long-term neurological sequelae in the Asia-Pacific region since the past decade [4]. In Taiwan, the largest and most severe outbreak occurred in 1998 with 78 deaths and 405 cases of neurological and cardiopulmonary complications [2]. Since then, EV71 infection has become endemic and continues to cause fatal outbreaks in Taiwan. Although EV71 has become an important pathogen for children, there are no vaccines and antiviral therapies currently available to control fatal infection due in part to the lack of understanding of viral pathogenesis. 

The roles of lymphocyte and antibody responses in EV71 infection are controversial. Insufficient lymphocyte response is shown to be a factor in increased morbidity and mortality, as young children are highly susceptible to infection and are likely to develop fatal symptoms, brainstem encephalitis and pulmonary edema. Moreover, significant reductions in lymphocyte numbers and responses are detected in the peripheral blood of patients with fatal symptoms [5, 6]. However, excessive immune response present during infection has been suspected to contribute to the pathogenesis of neurological symptoms. Some clinical studies find increased numbers of lymphocytes in the blood, cerebrospinal fluid, and CNS of patients with fatal symptoms [7–11]. Furthermore, a clinical report showed that a patient developed opsomyoclonus syndrome, an autoimmune disease [12]. The high titer of virus-specific antibody and the responsiveness of the condition to anti-inflammatory agents (corticosteroids) in this patient provide further evidence that there is an immunopathological aspect to this infection. Another clinical study also reports that corticosteroids, which mainly suppress T cells, improve the long-term neurological sequelae of patients [13]. Moreover, elevated levels of cytokines, such as interferon gamma, interleukin-1*β*, and interleukin-6 related directly or indirectly to lymphocyte response, are detected in the cerebrospinal fluid of patients with brainstem encephalitis [14, 15] and might contribute to neuroinflammation. The controversial issue regarding the roles of lymphocyte and antibody responses in EV71 infection needs to be resolved before development of effective vaccines and antiviral therapies to control fatal infection. 

The murine model has been used to study EV71 pathogenesis, as EV71 infection induces death and paralysis in mice as in humans [16]. Our previous study investigating the roles of lymphocyte and antibody responses in EV71 infection detected the presence of lymphocytes (B cells, CD4^+^ T cells, and CD8^+^ T cells) in the CNS of infected patients and mice [16]. In the present study, using lymphocyte-deficient mice, we found that B cells, but not CD4^+^ T cells, were required to prevent death in EV71-infected mice under the inocula of 8 × 10^6^–4 × 10^5^ plaque forming units (PFU)/mouse. This finding is inconsistent with the concept that CD4^+^ T cells are generally required for B cells to produce protective antibodies. We further investigated and found that mice deficient in CD4^+^ T cells produced neutralizing antibodies to reduce tissue viral loads and protect mice from EV71 infection.

## 2. Materials and Methods

### 2.1. Cell, Virus, and Mice

 A human muscle (rhabdomyosarcoma (RD)) cell line was maintained in medium according to the instructions of American Type Culture Collection. EV71 strain M2 was propagated and titrated in RD cells and used to infect mice as previously described [17]. Wild-type (C57BL/6J) mice and C57BL/6J-derived mice deficient in B cells (B6.129S2-*Igh*-*6* ^*tm1Cgn*^/J), CD4^+^ T cells (B6.129S2-*Cd4* ^*tm1Mak*^/J), or CD8^+^ T cells (B6.129S2-*Cd8a* ^*tm1Mak*^/J) were purchased from The Jackson Laboratory and bred under specific-pathogen-free conditions in the Laboratory Animal Center of our university. All mouse experiment protocols were approved by the Laboratory Animal Committee of National Cheng Kung University.

### 2.2. Infection of Mice

Nine-day-old mice were infected with 8 × 10^6^ or 4 × 10^5^ PFU/mouse of EV71 by oral inoculation as previously described [18]. Infected mice were monitored daily for signs of disease and survival. The disease score was graded as follows: 0, healthy; 1, ruffled hair; 2, weakness in hind limbs; 3, paralysis in single hind limb; 4, paralysis in both hind limbs; and 5 death. In separate experiments, mouse tissues were collected 5 days after infection to determine viral titers by plaque assay on RD cell monolayers as previously described [17]. 

### 2.3. Depletion of CD4^+^ T Cells in Mice

An antibody directed against mouse CD4^+^ T cells was purified from the ascitic fluid produced by hybridoma GK1.5 using Melon*™* Gel Monoclonal IgG Purification Kit (Pierce) according to the manufacturer's protocols and quantified using a spectrophotometer (Beckman). To deplete CD4^+^ T cells, wild-type mice were given one intraperitoneal injection of anti-CD4 antibody (100 *μ*g/mouse) or isotype-matched control antibody 2 days before inoculation with 8 × 10^6^ PFU/mouse of EV71. The efficacy of depletion was assessed by flow cytometry. In brief, mouse spleens were harvested, homogenized, and treated twice with buffer (0.14 M NH_4_Cl/0.017 M Tris, pH 7.2) to lyse red blood cells. The resulting cells were washed with RPMI medium containing 10% fetal bovine serum and stained with the phycoerythrin-conjugated control antibody (eBioscience) or antibody against mouse leukocyte antigen, CD4 (clone GK 1.5; eBioscience) or CD19 (clone 6D5; eBioscience) for 45 minutes on ice. The stained cells were analyzed using a FACSCalibur (BD Biosciences) with WinMDI software. 

### 2.4. Neutralization Assay

Mouse sera were collected 0, 7, and 11 days after infection to determine neutralizing titers as previously described [19]. Briefly, serial twofold dilutions of heat-inactivated serum were mixed with twofold 100% tissue culture infective doses of EV71. The serum and virus mixtures were incubated at 37°C for 1 hour before 1 × 10^4^ RD cells were added. The cultures were incubated for 3 more days and stained with crystal violet to observe cytopathic effect. The highest dilution of serum that protected RD cell monolayers from infection was recorded as the neutralizing titer.

### 2.5. Enzyme-Linked Immunosorbent Assay (ELISA)

EV71-specific ELISAs were performed by coating wells of 96-well plates with virus. Wells coated with virus were incubated with phosphate buffer saline containing 3% bovine serum albumin for 2 hours at room temperature to block nonspecific binding. Subsequently, serial twofold dilutions of serum samples harvested from mock- or EV71-infected mice were added to wells. After extensive washing, the bound antibody was detected by peroxidase-conjugated goat anti-mouse IgM (Calbiochem) or IgG (Chemicon) before developing color by adding 2,2′-azino-bis(3-ethylbenzothiazoline-6-sulfonic acid) (Sigma). Plates were read at OD_405_ by a microplate reader (Thermo LabSystems).

### 2.6. Antibody Treatment

CD4 T-cell-deficient mice were mock-infected or infected with 8 × 10^6^ PFU/mouse of EV71. Mouse sera were collected 7–10 days after infection, and the antibodies in mouse sera were purified by Melon*™* Gel Immunoglobulin Purification Kit (Pierce) according to the manufacturer's protocols and quantified using a spectrophotometer (Beckman). B-cell-deficient mice were given antibodies (100 *μ*g/mouse) purified from mock-infected or infected CD4 T-cell-deficient mice by intraperitoneal injection 1 day before and 1 day after as well as 1 day after infection with 8 × 10^6^ PFU/mouse of EV71.

### 2.7. Statistical Analyses

 Data are expressed as mean ± or plus standard error (SE) values. For statistical comparison, Kaplan-Meier survival curves were analyzed by log-rank test, disease score curves were analyzed by Wilcoxon signed-rank test, and viral titers were analyzed by Mann-Whitney *U* test. All *P* values are for two-tailed significance tests. A *P* value of < 0.05 was considered significant.

## 3. Results

### 3.1. Mice Deficient in CD4^+^ T Cells Are as Resistant to EV71 Infection as Wild-Type Mice, Whereas Mice Deficient in B Cells Are Highly Susceptible to EV71 Infection

**Figure 1:**
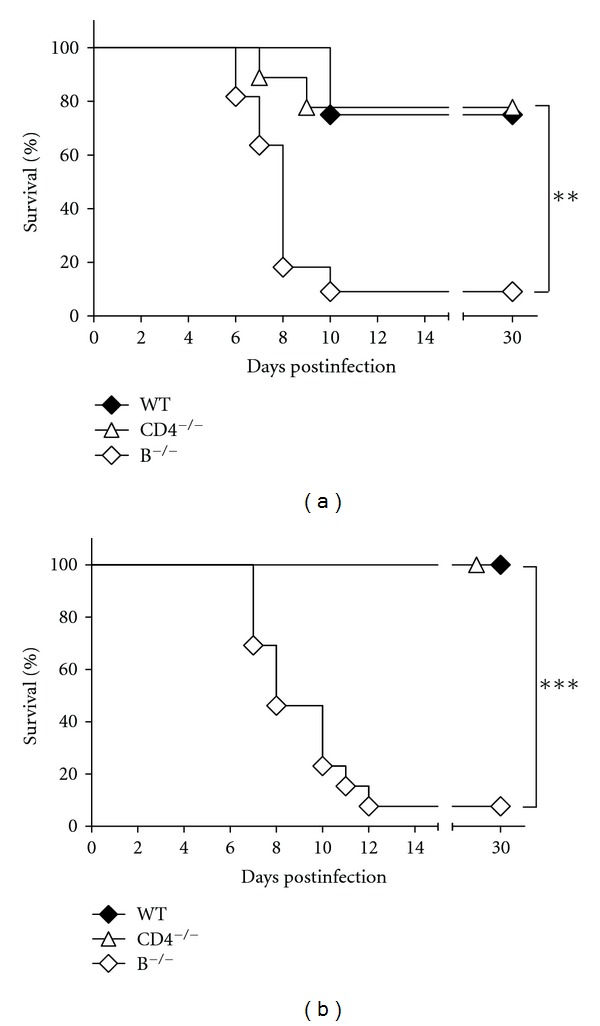
Infection of mice with EV71. (a) The survival rates of wild-type mice (WT; *n* = 8), CD4^−/−^ mice (*n* = 9), and B^−/−^ mice (*n* = 11) infected with 8 × 10^6^ PFU/mouse of EV71 are shown. (b) The survival rates of wild-type mice (WT; *n* = 12), CD4^−/−^ mice (*n* = 13), and B^−/−^ mice (*n* = 13) infected with 4 × 10^5^ PFU/mouse of EV71 are shown. Survival rates that are significantly different by the log-rank test are indicated as follows:  **P < 0.01;  ***P < 0.001.

Wild-type (C57BL/6J) mice and C57BL/6J-derived mice deficient in B cells or CD4^+^ T cells due to gene mutations were used for this study. We first evaluated the susceptibility of B-cell-deficient (B^−/−^) mice, CD4 T-cell-deficient (CD4^−/−^) mice, and wild-type mice to EV71 infection by inoculating mice orally with 8 × 10^6^ PFU/mouse of virus. By day 30 postinfection (p.i.), 78% (7 of 9) CD4^−/−^ mice and 75% (6 of 8) wild-type mice survived (Figure 1(a)). This was in stark contrast to infected B^−/−^ mice with a survival rate of 9% (1 of 11), which is significantly lower than those of CD4^−/−^ and wild-type mice (*P* < 0.01, log-rank test). We also tested mice infected with a much lower inoculum (4 × 10^5^ PFU/mouse; Figure 1(b)), which still caused death in almost all (12 of 13) B^−/−^ mice, but not in any CD4^−/−^ mice (*n* = 13) or wild-type mice (*n* = 12). Thus, B cells, but not CD4^+^ T cells, are essential to reduce EV71 lethality in mice infected with < 8 × 10^6^ PFU/mouse of virus. This finding is different from our previous report showing that the death rate of CD4^−/−^ mice was significantly higher than that of wild-type mice by about 40% after infection with a much higher dose (≥ 3 × 10^7^ PFU/mouse) of EV71 [16]. 

**Figure 2:**
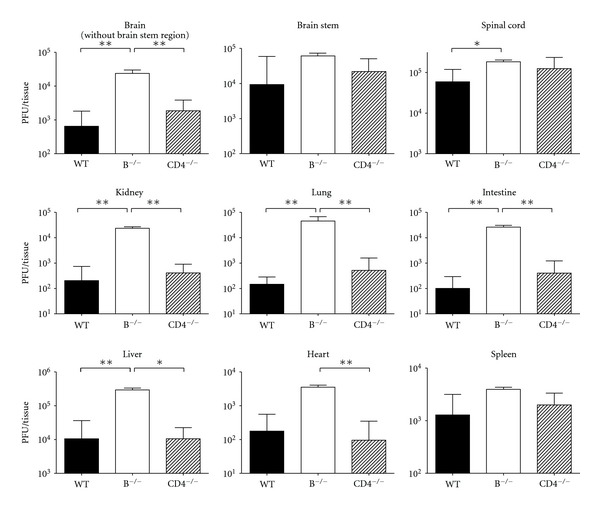
The tissue viral loads of EV71-infected mice. The viral titers in the indicated tissues of wild-type mice (WT; *n* = 6), B^−/−^ mice (*n* = 6), and CD4^−/−^ mice (*n* = 6) 5 days after infection are shown. The data shown are the mean values plus SE values (error bars). Viral titers that are significantly different by the Mann-Whitney *U* test are indicated as follows:  *P < 0.05;  **P < 0.01.

We next determined the tissue viral loads of mice inoculated with 8 × 10^6^ PFU/moue of EV71. Mouse CNS (the brain without the brain stem region, brain stem, and spinal cord) and peripheral tissues (the kidney, lung, intestine, liver, heart, and spleen) were harvested at day 5 p.i., the time point with abundant virus detected in tissues shown by our previous report [16]. In all the tissues examined, the mean viral titers of CD4^−/−^ mice were comparable to or slightly higher than those of wild-type (Figure 2). However, in all these tissues, the mean viral titers of B^−/−^ mice were all higher than those of CD4^−/−^ and wild-type mice with significant differences found in several vital tissues, the brain without the brain stem region, kidney, lung, intestine, and liver (*P* < 0.05, Mann-Whitney *U* test) by > 100- to 10-fold. Accordingly, B cells, but not CD4^+^ T cells, are required to reduce EV71 replication in tissues of mice infected with low doses of virus. 

### 3.2. The Resistance of CD4^−/−^ Mice to EV71 Infection Is Not due to Compensation of CD4 T-Cell Function by Other Immune Cells

One report showed that the peripheral CD8^+^ T-cell population in CD4^−/−^ mice is expanded [20]. To determine the importance of CD8^+^ T cells in protecting mice from EV71, we inoculated CD8^+^ T-cell-deficient (CD8^−/−^) mice with 4 × 10^5^ or 8 × 10^6^ PFU/mouse of virus. All eight CD8^−/−^ mice infected with 4 × 10^5^ PFU/mouse of virus survived, and 67% (4 of 6) CD8^−/−^ mice infected with 8 × 10^6^ PFU/mouse of virus survived, at rates similar to those of infected CD4^−/−^ and wild-type mice. Based on these results, the capacity of CD4^−/−^ mice to recover from EV71 infection in a manner similar to wild-type mice is not likely due to the compensation of CD4 T-cell function by CD8^+^ T cells. 

**Figure 3:**
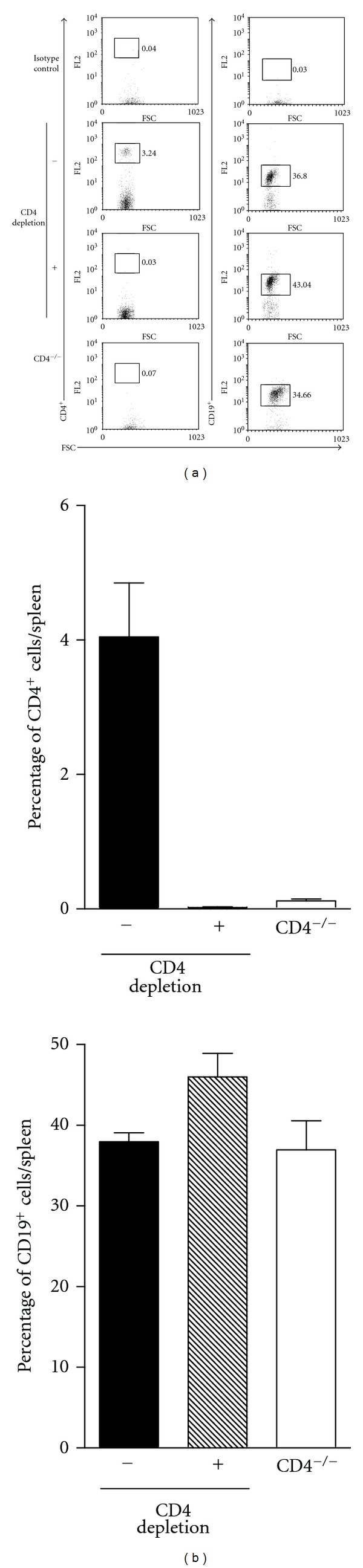
Anti-CD4 antibody treatment efficiently reduced CD4^+^ T cells in the spleen of wild-type mice. (a) The percentages of CD4^+^ T cells and CD19^+^ B cells in the spleens of wild-type mice treated with control antibody (− CD4 depletion) or anti-CD4 antibody (+ CD4 depletion) 7 days postinfection analyzed by flow cytometry are shown. CD4^−/−^ mice serve as a control for the assay. (b) The average percentages of CD4^+^ T cells and CD19^+^ B cells in spleens of the indicated mouse groups are shown. The data shown are the mean values plus SE values (error bars) of 3 spleens.

To further investigate the possibility of compensation of CD4 T-cell function by other immune cells in CD4^−/−^ mice, we depleted CD4^+^ T cells in wild-type mice with one intraperitoneal injection of anti-CD4 (GK1.5) antibody 2 days before EV71 infection. This depletion protocol has been shown to significantly reduce CD4^+^ T cells in the mouse spleen by > 95% 6 days after herpes simplex virus infection [21]. Consistent with the herpes simplex virus study [21], flow cytometry analysis showed that anti-CD4 antibody treatment efficiently reduced CD4^+^ T cells in the mouse spleen by > 99% 7 days after EV71 infection (Figure 3). Anti-CD4 antibody treatment did not affect the level of CD19^+^ B cells in the mouse spleen 7 days after EV71 infection. Depletion of CD4^+^ T cells did not cause death in any of five wild-type mice infected with 8 × 10^6^ PFU/mouse of virus. This result confirms that CD4^+^ T cells are not essential to reduce EV71 lethality in mice and also suggests that compensation of CD4 T-cell function by other immune cells in EV71-infected CD4^−/−^ mice is not likely. Our results also indicate that B cells might contribute to protecting CD4^−/−^ mice from EV71 infection.

### 3.3. Infected CD4^−/−^ Mice Produce Virus-Specific Neutralizing Antibodies, IgM and IgG

Although B cells can protect the host from viral infections by several mechanisms, including antigen presentation, cytokine production, and costimulation, the production of protective antibodies by B cells with the help from CD4^+^ T cells is generally thought to be critical. The antibody produced by B cells indeed plays a significant role in EV71 infection, as our previous report showed that treatment with the antibody purified from sera of immunized wild-type mice significantly reduced the lethality of infected B^−/−^ mice by reducing tissue viral loads [16]. 

**Figure 4:**
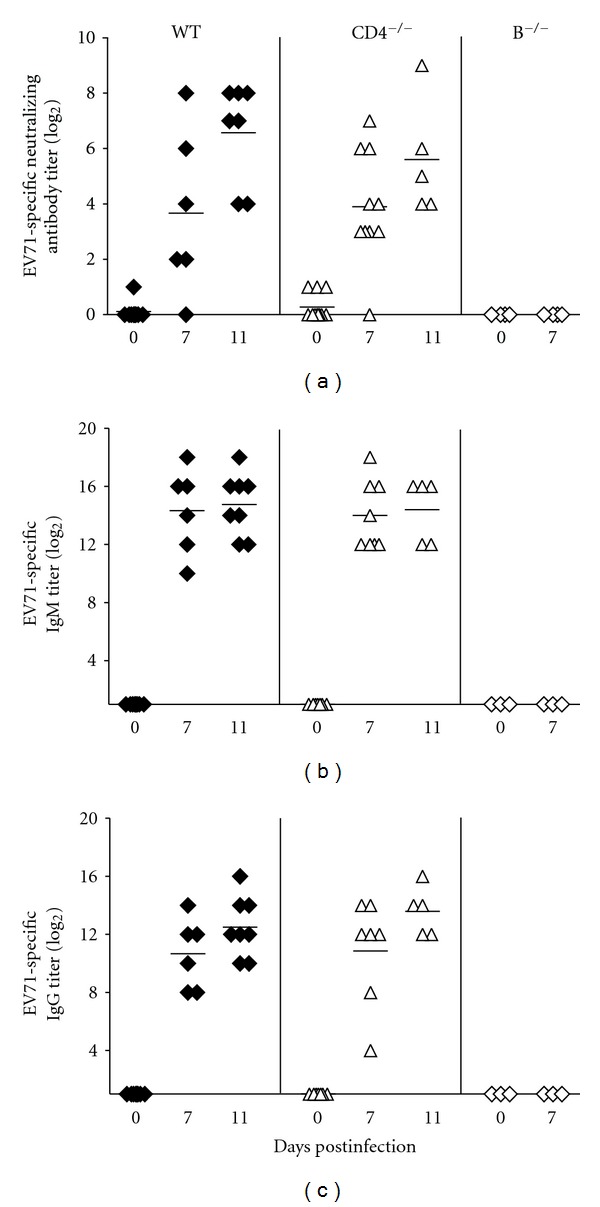
The antibody profile of EV71-infected mice. The titers of (a) neutralizing antibody, (b) EV71-specific IgM, and (c) EV71-specific IgG in sera of wild-type, CD4^−/−^, and B^−/−^ mice at the indicated times postinfection are shown. Each symbol on the scatter gram represents an individual sample, and the black horizontal line represents the mean value for each group.

We therefore investigated whether CD4^−/−^ mice could produce EV71-specific antibody with neutralizing activity. Sera were collected from infected mice at days 0, 7, and 11 p.i. and subjected to neutralization assay and ELISA. Neutralization assay showed that sera collected from wild-type, CD4^−/−^, or B^−/−^ mice at day 0 p.i. failed to neutralize EV71 (Figure 4(a)). Sera collected from wild-type and CD4^−/−^ mice, but not from B^−/−^ mice, at day 7 p.i. were able to neutralize virus. This is consistent with the survival result showing that, at this time point, the majority of B^−/−^ mice, but few wild-type or CD4^−/−^ mice, succumbed to death after infection. At day 11 p.i., the neutralizing antibody titers of both wild-type and CD4^−/−^ mice were further increased. The neutralizing antibody titers of CD4^−/−^ mice were slightly lower than those of wild-type mice from days 7–11 p.i.

ELISA detected EV71-specific IgM in sera of wild-type mice collected from days 7 and 11 p.i., but not at day 0 p.i., and the titers of EV71-specific IgM in sera of wild-type mice remained constant from days 7–11 p.i. (Figure 4(b)). EV71-specific IgM was also detected in sera of CD4^−/−^ mice with kinetics and titers comparable to those of wild-type mice from days 7–11 p.i. However, EV71-specific IgM was not detected in sera of B^−/−^ mice from days 0–7 p.i. 

EV71-specific IgG was also detected in sera of wild-type mice collected from days 7 and 11 p.i., but not at day 0 p.i., and the titers of EV71-specific IgG in sera of wild-type mice were increased from days 7–11 p.i. (Figure 4(c)). Similarly, EV71-specific IgG was detected in sera of CD4^−/−^ mice from days 7–11 p.i. with kinetics and titers comparable to those of wild-type mice, showing that the B cells of CD4^−/−^ mice are capable of switching the antibody class from IgM to IgG. Again, EV71-specific IgG was not detected in sera of B^−/−^ mice from days 0–7 p.i. 

### 3.4. The EV71-Specific Antibody from Infected CD4^−/−^ Mice Reduces EV71 Lethality in B^−/−^ Mice by Decreasing Tissue Viral Loads

**Figure 5:**
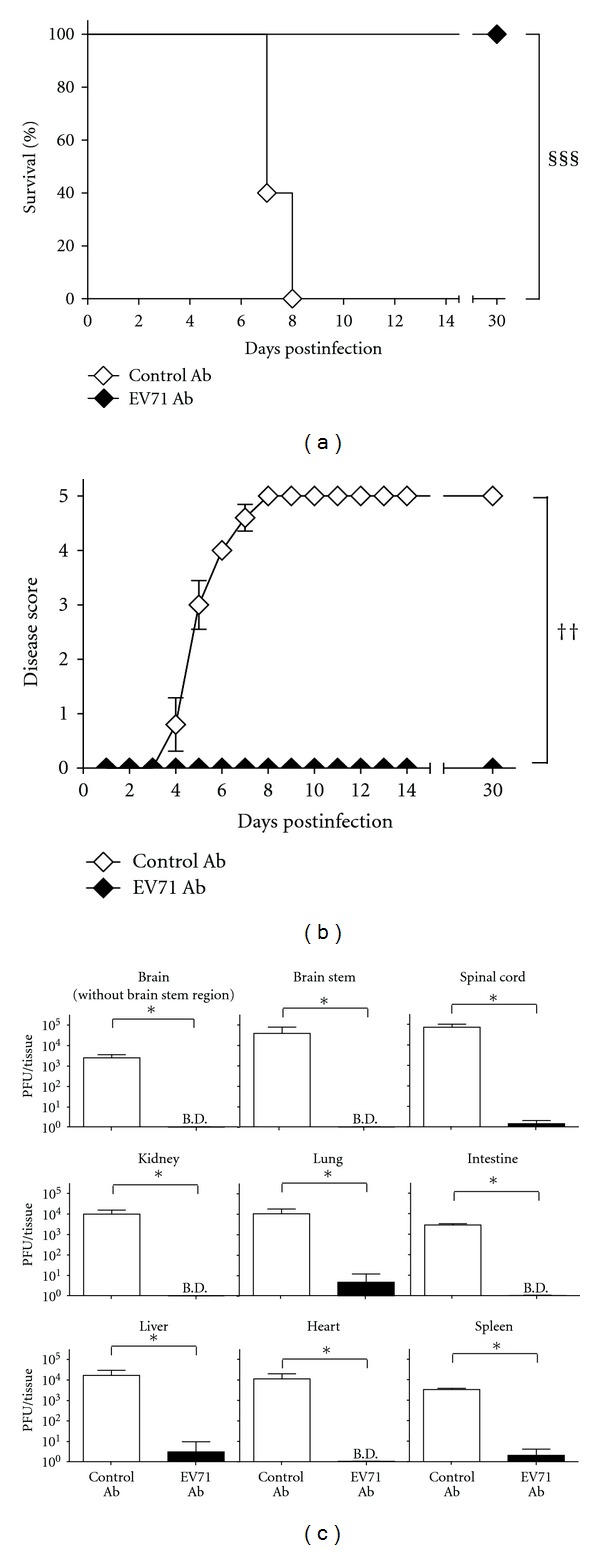
Treatment with the EV71-specific antibody from infected CD4^−/−^ mice reduces the lethality and disease severity of infected B^−/−^ mice by decreasing tissue viral loads. The (a) survival rates and (b) disease scores of infected B^−/−^ mice given the control antibody (control Ab; *n* = 5) from noninfected CD4^−/−^ mice or the EV71-specific antibody (EV71 Ab; *n* = 8) from infected CD4^−/−^ mice are shown. (c) The viral titers in the indicated tissues of B^−/−^ mice given control antibody (control Ab; *n* = 4–6) or EV71-specific (EV71 Ab; *n* = 4–6) antibody 5 days after infection are shown. The data shown in (b) and (c) are the mean values ± or plus SE values (error bars). Survival rates that are significantly different (*P* < 0.001) by the log-rank test are indicated (§§§). Disease scores that are significantly different (*P* < 0.01) by the Wilcoxon signed-rank test are indicated (††). Viral titers that are significantly different (*P* < 0.05) by the Mann-Whitney *U* test are indicated (∗).

Given that CD4^−/−^ mice were perfectly competent in fighting EV71 infection by reducing tissue viral loads as wild-type mice, whereas B^−/−^ mice were unable to do so, we postulated that the antibody produced by infected CD4^−/−^ mice might protect B^−/−^ mice from EV71 infection. To test this hypothesis, B^−/−^ mice were given intraperitoneal injections of the antibody purified from sera of infected CD4^−/−^ mice 1 day before and 1 day after inoculation with 8 × 10^6^ PFU/mouse of virus. As a control, the antibody purified from sera of noninfected CD4^−/−^ mice was used. After infection, all eight B^−/−^ mice given EV71-specific antibody survived, but all five B^−/−^ mice given control antibody died with a survival rate significantly lower than that of mice receiving EV71-specific antibody (*P* < 0.001, log-rank test; Figure 5(a)). Treatment with EV71-specific antibody also significantly and almost completely reduced not only the morbidity during acute infection but also the paralysis sequela during convalescence in infected B^−/−^ mice when compared with mice treated with control antibody (*P* < 0.01, Wilcoxon signed-rank test; Figure 5(b)). We also tested B^−/−^ mice given EV71-specific antibody after infection. However, all five infected mice given one dose of antibody at day 1 p.i. died. 

In separate experiments, we harvested the tissues of infected mice given antibodies 1 day before and 1 day after infection to determine viral loads. In all the tissues examined, the CNS (the brain without the brain stem region, brain stem, and spinal cord) as well as peripheral tissues (the kidney, lung, intestine, liver, heart, and spleen), the mean viral titers of B^−/−^ mice given EV71-specific antibody were significantly lower than those of B^−/−^ mice given control antibody (*P* < 0.05, Mann-Whitney *U* test) with > 1,000-fold difference at day 5 p.i. (Figure 5(c)). These results collectively show that the CD4 T-cell-independent antibody response protects mice from EV71 infection by reducing tissue viral loads.

## 4. Discussion

This is the first report to show the essential role of CD4 T-cell-independent antibodies in protecting mice from EV71 infection by reducing tissue viral loads. These results lead to a better understanding of how the host elicits antibody response to combat fatal EV71 infection. The present study also has implications for host susceptibility to EV71 infection and for the use of vaccines and antibodies to reduce mortality in susceptible hosts. 

Here we show that B cells produce protective antibodies with neutralizing activity against EV71 infection in CD4^−/−^ mice. Marginal zone B cells located in the marginal zone of spleens and B1 B cells located in the peritoneal and pleural cavities are two subsets of B cells classified as innate-like B cells capable of secreting IgM and IgG with neutralizing activity upon stimulation with T-cell-independent antigens in mice [22–25]. Viral infections have been shown to stimulate innate-like B cells to produce CD4 T-cell-independent antibodies by inducing extensive cross-linking of B cell receptors, by engagement with toll-like receptors, or by producing cytokines [26]. In CD4^−/−^ mice, EV71 infection might activate innate-like B cells to produce protective CD4 T-cell-independent antibodies through these pathways. The capsid of vesicular stomatitis virus with approximately 1300 G protein monomers is shown to serve as a T-cell-independent antigen to activate B cells by extensively cross-linking B cell receptors [26, 27]. Similar cross-linking may occur during EV71 infection, as EV71 capsid is composed of 60 identical units [28]. 

EV71-infected CD4^−/−^ mice could produce neutralizing antibodies with reduced titers, when compared with infected wild-type mice. Our previous report showed that 25 *μ*g of antibodies from immunized wild-type mice was sufficient to protect B^−/−^ mice infected with ≥ 3 × 10^7^ PFU/mouse of EV71 even when the antibody was given 1 day p.i. [16]. In the present study, our test found that 100 *μ*g of antibodies from infected CD4^−/−^ mice was the minimum amount required to protect B^−/−^ mice infected with 8 × 10^6^ PFU/mouse of virus and that the antibody treatment given 1 day p.i. was ineffective. These results indicate that although CD4^+^ T cells might not be required for B cells to produce protective antibodies or switch immunoglobulin class from IgM to IgG, they do help B cells to produce high titers of neutralizing antibodies. 

In this study, we reveal that B cells play a much more important role than CD4^+^ and CD8^+^ T cells in protecting mice from EV71 infection when we use low doses of virus to infect lymphocyte-deficient mice. This finding is similar to previous clinical reports showing that patients with agammaglobulinemia, but normal T-cell function, are highly susceptible to the CNS infection induced by another enterovirus, echovirus [29, 30]. There are few studies on why some individuals are highly susceptible to EV71 infection. Our results showing the significant role of CD4 T-cell-independent antibodies in EV71 infection suggest that it might be worth investigating whether susceptible patients might have defects in antibody response. 

There are no effective vaccines currently available to control fatal EV71 outbreaks. In our present and previous studies, we show that virus-specific antibodies with neutralizing activity given before infection protect susceptible mice from infection [16]. Therefore, vaccines should have great potential to control fatal outbreaks. There are no specific antiviral therapies currently available to treat patients with fatal symptoms. Intravenous immunoglobulin, which has several anti-inflammatory properties and often contains low titers of neutralizing antibodies to enteroviruses, has been a mandatory treatment for EV71 patients with neurological symptoms in Taiwan, because it has been shown to improve the conditions of patients infected with other enteroviruses, coxackievirus B and echovirus [7, 29, 31, 32]. Our previous study showed that virus-specific antibodies with neutralizing activity, but not control antibodies, given after infection protect susceptible mice from infection [16]. Therefore, virus-specific antibodies with high neutralizing titers may have great potential and might be better than intravenous immunoglobulin for reducing mortality in infected patients. The results from our studies are particularly important in view of the fact that the emerging pathogen, EV71, is very likely to continue to pose serious risk to large numbers of young children in the future.
